# Delayed reversal of methanol-induced blindness in an adult by a combination of erythropoietin and a high dose of methylprednisolone: a case report

**DOI:** 10.1186/s13256-023-03899-w

**Published:** 2023-04-20

**Authors:** Fatemeh Vafapour, Sajad Jahangiri-Mehr, Sajad Hassanzadeh

**Affiliations:** 1 Department of Internal Medicine, School of Medicine, Yasuj University of Medical Sciences, Shahid Dr. Ghorban Ali Jalil Street, Yasuj, Iran; 2 Department of Ophtalmology, School of Medicine, Yasuj University of Medical Sciences, Yasuj, Iran

**Keywords:** Methanol toxicity, Blindness, Erythropoietin

## Abstract

**Background:**

The clinical manifestation following methanol toxicity accounts for a life-threatening problem that contributes to metabolic disorders, neurological complications, blindness, and even death. There is no completely effective treatment to retain the patient’s vision. Herein, we apply a new therapeutic strategy for the recovery of bilateral blindness in a patient who had ingested methanol.

**Case presentation:**

A 27-year-old Iranian man with complete bilateral blindness was referred 3 days after accidental ingestion of methanol to the poisoning center at Jalil Hospital, Yasuj, Iran, in 2022. After taking his medical history, performing neurologic and ophthalmologic examinations, and routine laboratory tests, ordinary management was undertaken and counterpoisons were given for 4–5 days; however, the blindness did not reverse. Following the 4–5 days of unsuccessful standard management, he was given ten doses of subcutaneous erythropoietin 10,000 IU/12 hours twice daily, folinic acid 50 mg/12 hours, and methylprednisolone 250 mg/6 hours for 5 days. After five days, vision of both eyes recovered, reaching 1/10 in the left and 7/10 in the right eye. He remained under daily supervision until his release from the hospital, and he was discharged from the hospital 15 days post admission. In outpatient follow-up, his visual acuity was improved without having any side effects at 2 weeks after discharge.

**Conclusion:**

A combination of erythropoietin and a high dose of methylprednisolone were useful for relieving the critical optic neuropathy and improved the optical neurological disorder following methanol toxicity.

## Background

Vision loss is a major consequence of alcohol consumption in people with alcoholism. According to the Global Burden of Disease Study, consumption of alcohol is the seventh-highest risk factor for disability-adjusted life years (DALYs), and leads to 2.84 million deaths worldwide [1]. Intake of about 14 g of poured alcohol in any form is defined as an alcohol drink [2]. Gastric distress, cardiovascular disease, diabetes, liver damage (cirrhosis), and bilateral vision loss, are the main manifestation of alcohol intake [3, 4].

Toxic neuropathy following alcohol consumption is defined as a bilateral progressive vision loss resulting in papillomacular bundle damage and cecocentral and central scotoma. Methanol is an alcohol derivative, which is colorless and similar to ethanol in taste and smell; therefore, accidental ingestion of methanol is common, and has serious side effects including blindness, neurological dysfunction, metabolic disorders, and even death [5]. The initial diagnosis is usually based on an ocular examination and evaluation of serum vitamin B12 and folate levels [6].

The basic intervention includes changes in diet, administration of intravenous vitamin B12, and folate supplements, along with hemodialysis, and 4-methylpyrazole for methanol toxification inhibition [7].

Erythropoietin has neuro-reformative, neuro-protecting, antiinflammatory, and antioxidant effects. While the mechanism of action is not absolutely clear, it is assumed to prevent apoptosis and assist in the regeneration of impaired neurons [8]. Furthermore, administration of erythropoietin is one of the best management strategies for prolonged neuro-progressive complications such as Alzheimer’s disease, amyotrophic lateral sclerosis, and Parkinson’s disease [9].

Here we report a delayed reversal of methanol-induced blindness in an adult by a combination of erythropoietin and a high dose of methylprednisolone.

## Case presentation

A 27-year-old Iranian healthy young man with no history of any significant medical complications but with a history of alcohol consumption was referred to the emergency department of Jalil Hospital, Yasuj, Iran. He presented with vomiting, diarrhea, and vision blurriness 3 days before the referral to the hospital. He did not give any information regarding the consumption of alcohol and had only received some supportive, symptomatic treatment. He referred to the emergency when he became completely blind and had no light perception (NLP).

In the emergency room, vitamin B6 and folic acid was ordered following detection of ethanol 20%, then hemodialysis was performed for detoxification for 4 hours. The patient’s body weight was 74 kg and other initial factors including heart rate, blood pressure, body temperature, and breathing rate were measured before and after hemodialysis (Table 1). The patient was referred for ophthalmological investigation, where he was diagnosed as being completely blind due to mydriasis in both pupils and with no light perception. After 3 days of standard treatment, no changes in his vision were observed.

**Table 1:** Clinical observations at the time of referral and after hemodialysis

Clinical observation	Clinical observation before hemodialysis	Clinical observation after hemodialysis	Clinical observation 3 days after hemodialysis
Weight	74 kg	74 kg	72 kg
Blood pressure	135/90 mm/Hg	150/90 mm/Hg	140/90 mm/Hg
Temperature	37.1 °C	37 °C	37.2 °C
Heart rate	89 beats/minute	78 beats/minute	70 beats/minute
Breathing rate	20 breaths/minute	18 breaths/minute	18 breaths/minute
Lactate	1.4 mmol/L	1.2 mmol/L	1.2 mmol/L
PH	7.30	7.34	7.35
HCO_3_	22.8	24	23
PCO_2_	38	41	37
Creatinine	1.1	1	1.15
Light perception	No sense	No sense	No sense

*PaCO*_*2*_ Partial pressure of carbon dioxide; *HCO*_*3*_ Bicarbonate; *pH* This measures the balance of acids and bases in your blood

**Fig. 1:**
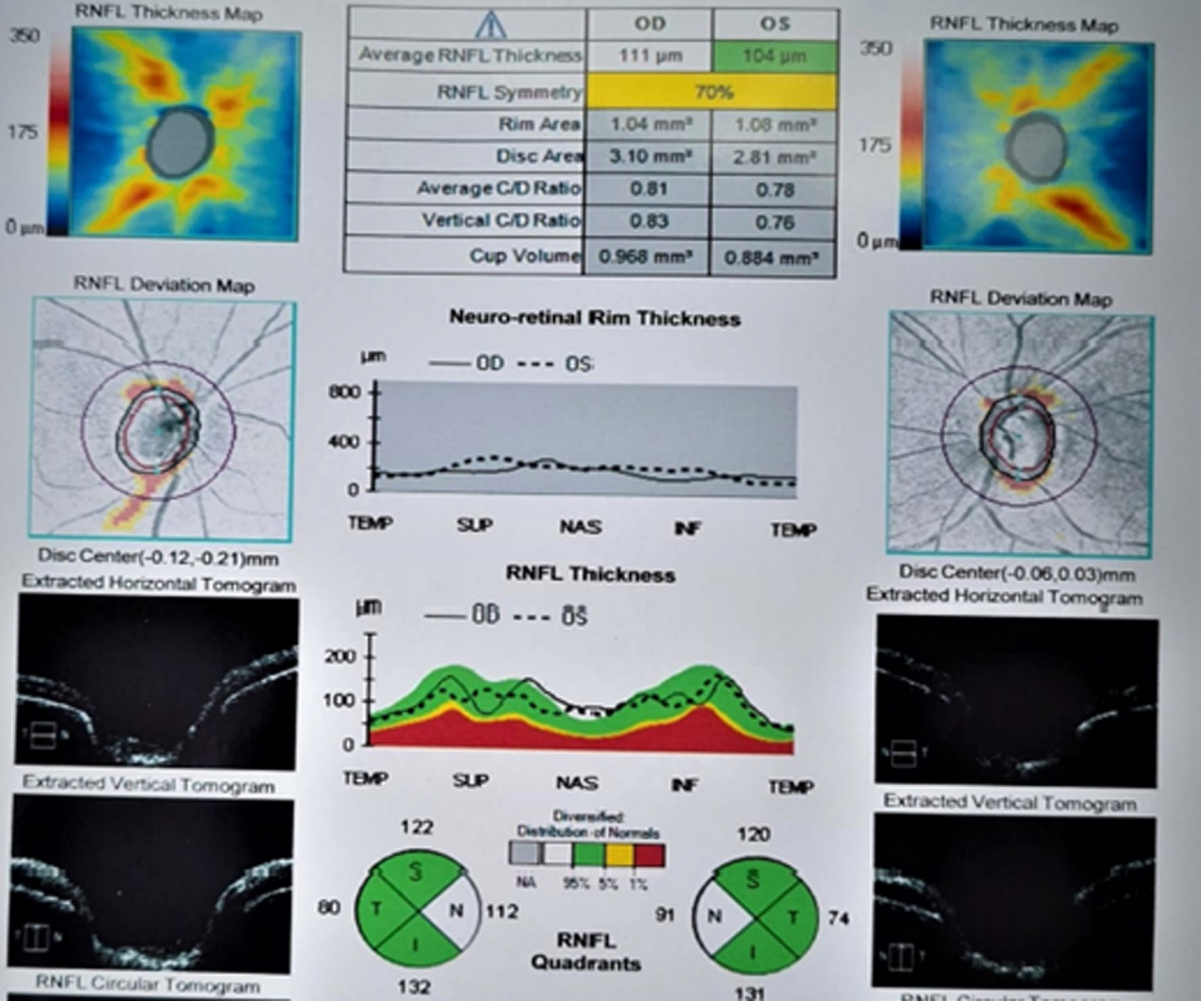
Spectral-domain OCT retinal nerve fiber layer thickness 5 days after toxicity. The thickness of the retinal nerve fiber layer was normal and decreased after toxicity. More than 80% cupping of the optic nerve head was observed after intervention. *OCT* Examination of the retinal layer was normal on admission and two weeks later

The following medical intervention was applied to improve his sight: intravenous administration of folinic acid 50 mg/12 hours, methylprednisolone 250 mg/6 hours, and erythropoietin (EPREX) 10,000 IU/12 hours, for 5 days. Following this new therapy, he developed light perception. Two days after administration of the new therapy, he could distinguish light perception and hand motion movement . On the third day, he could count fingers and numbers, and had visual activity, and on the fifth day, his vision had improved (7/10 in the right and 1/10 in the left eyes). According to the optic nerve head (ONH) and retinal nerve fiber layer (RNFL) analysis, the thickness of the nerve fibers was normal, while the thickness of neuro retina fibers was decreased. The characteristic cupping of the ONH was above 80% and there had been no sign of cupping at the time of the referral (Fig. 1).

## Discussion

Methanol toxicity is a prominent cause of blindness in the twentieth century, although the rate of toxicity in developed countries is less than in developing countries [7]. Methanol toxicity symptoms are observed after 12–24 hours following ingestion, starting with gastrointestinal symptoms such as abdominal pain, vomiting, and diarrhea, along with neuro-optic complications [5]. The ocular manifestation consists of difficulty and pain in eye movement, blurred vision, reduced sharpness of sight, photophobia, and optic disc edema with twisted retinal vessels [6]. The clinical manifestation occurs as a result of formaldehyde and formic acid metabolites, which also contribute to the difficulty in breathing due to acidosis. Furthermore, if the metabolites accumulate at a higher percentage, it can lead to coma, respiratory system failure, and eventually death [7].

To the best of our knowledge, treatment strategies include neutralizing the metabolite formation through inhibition of the alcohol dehydrogenase enzyme, along with hemodialysis to remove the remaining metabolites [10, 11] According to a MEDLINE systematic review from 1966 to 2010, ethanol was used for most people with alcohol toxicity (80%), while 16% were treated with fomepizole. According to the American Association of Professional Code Center (AAPC) National Poison Data System Database, fomepizole was administered in 90% of patients with methanol and ethanol toxicity [12]. Fomepizole can only eliminate the methanol metabolites from the lungs and kidneys, with a slow mean half-life of 54 hours [13], while hemodialysis rapidly removes the methanol, with 400 ml/minute of blood flow in 4 hours at a dialysis flow of 800 ml/minute [14]. Administrating both fomepizole and hemodialysis has recently been recommended in cases with no evidence of optical complications [15]. Due to the lack of access to fomepizole in Iran, we could not administer it, so we used 20% ethanol as an alternative [15]. We applied the hemodialysis to reduce the symptoms of toxicity, but we did not observe any significant changes in the clinical manifestation, especially in the recovery of bilateral blindness. The occurrence of optic neuropathy resulting from methanol toxicity accounts is a major challenge because the exact pathology is not fully understood, therefore, an effective medicine is not yet available [16]. A study conducted by Sivilotti *et al*. found that administration of folinic acid could prevent the formation of toxic metabolites but did not have any effect on methanol toxicity [17]. Also, the intravenous injection of steroids could have a beneficial effect on blindness following methanol toxicity; hence, the interval between ingestion and medical intervention is crucial [18]. Shukla *et al*. applied intravenous methylprednisolone to 17 patients with methanol toxicity and observed a better visual outcome (88/2%), decreased disc edema, and clearance of disc margin after 3 months. Hand motion perception was observed in one patient at the time of intervention and then at 1 month after the intervention. One patient had a perception of light at the time of intervention in both eyes, whilst two patients had light perception in both eyes while others did not have any light perception after 3 months, and two patients had counting finger perception after using methylprednisolone. Finally, visual acuity was about 6/36 in both left and right eyes in all 17 patients [3]. Herein, we applied a new medical intervention using methylprednisolone, recombinant erythropoietin, and folinic acid for patients with complete bilateral blindness, and observed a relative recovery of eyesight and visual acuity almost 20 days after discharge from the hospital, with 10/10 visual acuity in both left and right eyes. Furthermore, the thickness of the retinal nerve fiber and the optic nerve head (ONH) normalized after administration of this treatment, and a sign of cupping increased up to 80%. Therefore, we suggest applying this medical protocol to patients with bilateral blindness following methanol toxicity, and have approved the efficiency of this therapeutic protocol on a large scale.

## Conclusion

This is the first report of administrating a combination of methylprednisolone, recombinant erythropoietin, and folinic acid for recovery of sight complications following ingestion of methanol. The application of these medicines decreased the time of hospitalization and accelerated the recovery of a patient with bilateral eye blindness, in whom the improvement of visual acuity was recovered to approximately its normal range.

## Data Availability

The datasets used and/or analyzed during the current study are available from the corresponding author on reasonable request.
